# Balancing ACT: evaluating the effectiveness of psychoeducation and Acceptance and Commitment Therapy (ACT) groups for people with bipolar disorder: study protocol for pilot randomised controlled trial

**DOI:** 10.1186/s13063-018-2789-y

**Published:** 2018-08-13

**Authors:** Emma O’Donoghue, Abigail Clark, Matthew Richardson, John Hodsoll, Sunil Nandha, Eric Morris, Fergus Kane, Deirdre O’Keeffe, Lucy Butler, Suzanne Jolley

**Affiliations:** 1 South London and Maudsley NHS Foundation Trust, London, SE5 8AZ UK; 2 Department of Biostatistics, King’s College London, Institute of Psychiatry, Psychology and Neuroscience, London, SE5 8LY UK; 3 LaTrobe University, Melbourne, Australia; 4 Department of Psychology, King’s College London, Institute of Psychiatry, Psychology and Neuroscience, London, SE5 8AF UK; 5 South London and Maudsley NHS Foundation Trust, 308-312 Brixton Road, London, SW9 6AA UK

**Keywords:** Bipolar disorder, Community mental health, Acceptance and Commitment Therapy (ACT), Psychosis, Psychoeducation

## Abstract

**Background:**

Bipolar disorder is a chronic and disabling psychiatric condition, characterised by recurrent episodes of mania, hypomania and depression. It places a heavy burden on sufferers and families, with high societal and healthcare costs. Many service users with a diagnosis of bipolar disorder also experience prominent psychotic symptoms, with differential diagnoses of schizoaffective disorder, and relapses characterised by repeated manic psychotic episodes and grandiosity. Such presentations require specific adaptations to standard bipolar disorder interventions in order to address their psychosis, alongside mood regulation, with a particular emphasis on impulsivity, irritability, disinhibition and elation. The Balancing ACT study aims to evaluate an innovative group intervention combining Acceptance and Commitment Therapy and psychoeducation approaches (ACT/PE) with individuals experiencing bipolar disorder and/or symptoms within community psychosis services.

**Methods:**

The Balancing ACT study is a randomised controlled trial comparing Balancing ACT groups (ACT/PE) plus routine care to routine care alone. Balancing ACT (ACT/PE) comprises ten group sessions, each lasting 2 hours, delivered weekly. The primary outcome is psychological wellbeing; secondary outcomes are mental health relapses (measured by service use averages for the 12 months pre baseline and 3 months post baseline). We will also measure mood, distress, recovery and psychological change processes. Participants will be randomised in a 1:1 ratio, after baseline assessment. Outcomes will be assessed by trained assessors blind to treatment condition at 0, 10 and 14 weeks. Recruitment began in April 2017 and is on-going until the end of October 2017.

**Discussion:**

The Balancing ACT study will contribute to the currently limited evidence base for psychological interventions for people experiencing bipolar disorder and/or symptoms in the context of community psychosis services.

**Trial registration:**

ISRCTN73327972. Registered on 27 March 2017. Balancing ACT: evaluating the effectiveness of psychoeducation and Acceptance and Commitment Therapy (ACT) groups for people with bipolar disorder.

**Electronic supplementary material:**

The online version of this article (10.1186/s13063-018-2789-y) contains supplementary material, which is available to authorized users.

## Background

Bipolar disorder is a chronic and disabling psychiatric condition, characterised by recurrent episodes of mania, hypomania and depression. It places a heavy burden on sufferers and families, and has high societal and healthcare costs [1, 2]. An estimated 2 to 6% of the general population in the UK meet the criteria for bipolar disorder, costing £5.2 billion annually in healthcare and lost employment [3]. Many of the service users with a diagnosis of bipolar disorder also experience prominent psychotic symptoms, with differential diagnoses of schizoaffective disorder, and relapses characterised by repeated manic psychotic episodes and grandiosity. Such presentations require specific adaptations to standard psychological interventions for bipolar disorder in order to address psychosis, alongside mood regulation, with a particular emphasis on impulsivity, irritability, disinhibition and elation [2].

The main treatment offered for bipolar disorder is medication [2]. Despite this, many individuals continue to experience residual symptoms, even when they are fully concordant with medication, with rates of relapse of between 40 and 60% [2, 4, 5]. Psychological therapies were previously thought to add little to the treatment of severe mental illness. However, psychological interventions for people with psychosis are now firmly established as clinical and cost-effective interventions, and, although the move away from a predominantly medical approach has been more recent for bipolar disorder, there is increasing evidence that patients benefit from high-quality psychological therapies, with clinical and cost effects, as well as improving the quality of life of service users and their families [6]. The National Institute for Health and Care Excellence [2] guidelines recommend up to 16 sessions of individual cognitive behaviour therapy (CBT) including psychoeducation and self-management/relapse prevention for bipolar disorder and the same duration of intervention for people with psychosis, incorporating self-monitoring, coping strategies, distress-reduction and improving functioning. Family Interventions (FI) and psychosocial/vocational support are also recommended for both bipolar disorder and psychosis.

A principal goal of psychoeducation for bipolar disorder is to provide accurate and reliable information, as well as teaching early recognition and self-management skills and facilitating service users to make informed decisions about the management of their own mental healthcare within the context of a collaborative working relationship with their clinical team [7]. Psychoeducation is usually facilitated in a group format and has also been found to be helpful for family members and caregivers of people with bipolar disorder, by reducing relapses and increasing time spent well [7, 8].

Psychoeducation for service users with bipolar disorder with mania and psychosis symptoms requires accurate and detailed information about psychosis, recovery, and a particular focus on symptoms of elevated mood and grandiosity. Traditional approaches to psychoeducation (e.g. teaching people about the medical nature of their condition) alone for people with psychosis have been found to be ineffective [9]. This could possibly be due to the difficulty of establishing a shared understanding between the service user and the clinical team in the context of differing levels of insight. Psychoeducation for this group must accommodate these specific engagement issues to develop individually tailored, validating and normalising understandings that do not inadvertently discredit personally valued unusual experiences and beliefs. Psychoeducational material of this kind has yet to be formally evaluated.

Acceptance and Commitment Therapy (ACT) is an emerging psychological intervention which facilitates self-management and acceptance of difficulties, and promotes a focus on valued personal goals, in the face of adversity [10]. ACT also promotes social inclusion by shifting the service user’s focus from symptom control to connecting with personal values and participating in life. The central tenets of ACT, and its empowering, equitable, open and collaborative therapeutic stance, therefore fit it well to a recovery ethos. Unlike many psychological interventions, ACT relies on a philosophy which can be readily trained, and a recent pilot study by our group in South London and Maudsley NHS Foundation Trust, funded by the Maudsley Charity, demonstrated that co-facilitators could learn to deliver ACT by co-facilitating groups over a 4-week course of therapy (Jolley S, Johns L, O’Donoghue E, Oliver J, Khondoker M, Byrne M, Butler L, De Rosa C, Sim F, Morris EM: A randomised controlled trial of group acceptance and commitment therapy for patients and caregivers in psychosis services, Submitted).

ACT has shown good preliminary evidence of effectiveness for people with psychosis [11, 12] and ACT trials have contributed to the recommendations of the NICE guidance [13]. ACT has recently been piloted for people with bipolar disorder who experience co-existing anxiety and found to be effective [14]. However, group ACT and psychoeducation interventions for people with bipolar disorder have not yet been evaluated in a randomised controlled trial (RCT). ACT’s transdiagnostic nature fits well with mood instability alongside psychotic symptoms: ACT aims to target transdiagnostic processes, and recent studies indicate the potential effectiveness of ACT across a broad spectrum of severe mental health conditions, including mood instability and psychotic symptoms [11, 12]. ACT frames values, in particular, as remaining relatively constant throughout a person’s life: mood episodes and other problematic symptoms lead to the person losing touch with their values, rather than fundamentally changing them. During depressive, hypomanic and manic episodes, it is common for people to engage in activities that are contrary to their normal values and/or to drop activities that are in line with such values [15]. Such changes may be driven, maintained and amplified by cognitive distortions, including mood-consistent altered perceptions of the future [15, 16], as well as experiential avoidance of painful emotions, and reduced meta-cognitive awareness. An explicit focus on values aims to help people stay in touch with said values during prodromal mood states, and thus remain engaged with ‘value-driven action’, helping prevent escalation into mania or descent into depression.

The values component of ACT helps people to get in touch with chosen life directions, which will be present regardless of an individual’s mood state; values provide a source of reinforcement and goal-directed activity independent of mood, as well as having elements that are recovery-consistent. Accordingly, recent studies indicate the potential for effectiveness across a broad spectrum of severe mental health conditions [11, 12].

### Study aims

We plan to carry out a pilot RCT to test out the potential feasibility and clinical and cost-effectiveness of our Balancing ACT groups incorporating ACT and psychoeducation (ACT/PE), as an adjunct to routine care (treatment as usual, TAU), in improving psychological wellbeing in adults (aged 18 years or over) with a diagnosis of bipolar disorder and/or bipolar symptoms in community psychosis services. The Balancing ACT (ACT/PE) intervention plus TAU condition will be compared to TAU alone. Service users allocated to TAU will be offered the Balancing ACT intervention after completing the final study assessment.

The specific research questions to be addressed are:

1. Are primary and secondary clinical outcomes for people with bipolar disorder and/or bipolar symptoms in psychosis community services potentially improved by the addition of ACT/PE groups? If so, to what extent, and with what likely variability in effect size?
2. Do the ACT/PE groups have a potential positive impact on mood, distress, recovery and psychological processes of change?
3. Is the ACT/PE intervention potentially cost-effective?
4. Is it feasible to add ACT/PE approaches to bipolar disorder psychological treatments?

## Methods

### Participants and setting

We aim to recruit 36 adults aged 18 years or over, presenting to the Promoting Recovery Service Community Mental Health Teams of the Psychosis Clinical Academic Group of the South London and Maudsley NHS Foundation Trust (SLaM) within King’s Health Partners. Ten teams, from the inner-London boroughs of Lambeth and Southwark are participating. The sample will include service users with diagnoses of bipolar affective disorder and schizoaffective disorder. A recent local audit showed that of 1344 adults currently treated in SLaM with a diagnosis of bipolar disorder, more than half (*N* = 823) are being treated within psychosis services, with around 200 in each borough (13–20% of the caseload). As such, we expect recruitment to be reasonably straightforward.

The proposed sample size, while representing only a small proportion of the caseload, is in line with recommendations for the number of participants required to estimate variance in outcomes and is, therefore, appropriate for an early stage feasibility study. (24 to 50 participants [17, 18]). The feasibility study is designed to feed into a second larger pilot study, which will benefit from feedback received in the current study, and allow consideration of the representativeness of the study sample.

#### Inclusion and exclusion criteria

Inclusion criteria are: presenting to, and being treated under, Lambeth or Southwark Promoting Recovery Services (the services see working age adults, aged 18 years or over); diagnosis of bipolar disorder and/or bipolar symptoms; being available for the study duration; sufficient English language ability to be able to complete assessment measures and participate in therapy, without interpreter support (as this cannot feasibly be provided in a group context). Exclusion criteria are: inability to remain in a group setting, attend and understand and interact for up to 2 h; considered by the treating team to lack capacity to consent. This will mean that people with acute extremes of mood state are unlikely to meet the inclusion criteria, although an extreme mood state will not be an exclusion criterion in itself.

### Study design

**Fig. 1:**
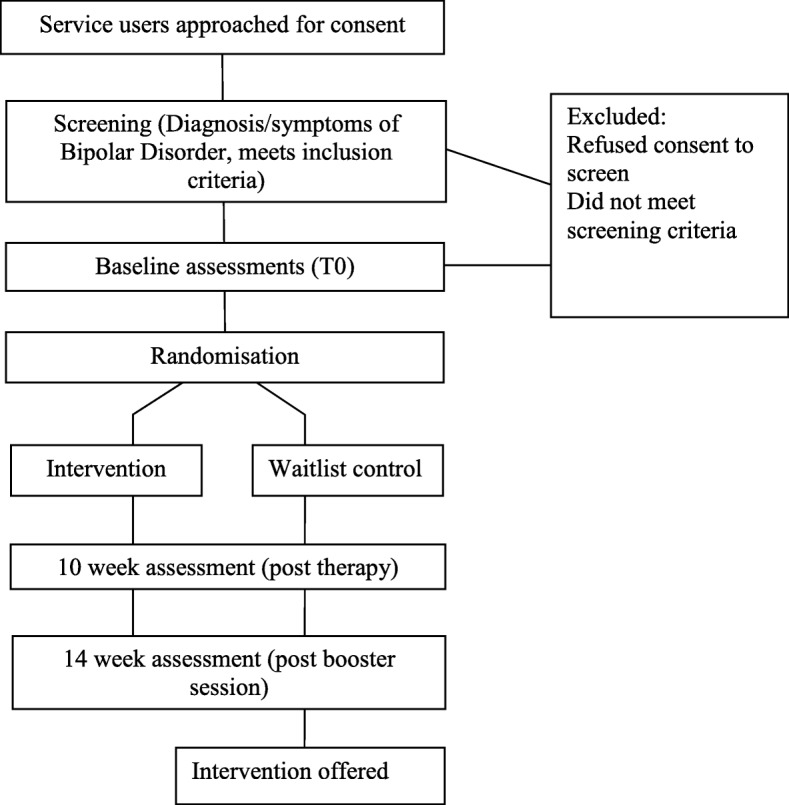
Balancing ACT: evaluating the effectiveness of psychoeducation and Acceptance and Commitment Therapy (ACT) groups for people with bipolar disorder: study protocol for a pilot randomised controlled trial. Study design

Balancing ACT is an RCT with random allocation to one of two arms, comparing our active intervention (ACT/PE + TAU) to routine care alone (TAU). TAU will be delivered without interference in both conditions and includes care coordination, medication, and practical/emotional support for the individual. We will record what is delivered as TAU. Assessments will take place at baseline (0 weeks), 10 weeks (post therapy) and 14 weeks (post-booster session). After 14 weeks, TAU participants will be offered the intervention. Trained research workers will complete assessments with participants. Baseline assessments will be carried out prior to randomisation; 10- and 14-week assessments will be arranged by the trial research worker, but carried out by a different assessor, who will be blind to treatment allocation. Service measures [19] will be completed for the 12-month period before baseline and 3 months after the final baseline assessment. The study design is illustrated in Fig. 1.

### Procedure

#### Recruitment

We will recruit directly from Promoting Recovery Services in Lambeth and Southwark boroughs. Adults presenting with bipolar disorder and/or symptoms will be invited by their team to find out more about the study, and, if agreeing, will be contacted by the Balancing ACT study researcher. Telephone conversations and meetings will take place as needed to discuss the study. Participants will be provided with an information sheet and consent form. If an individual consents to participate, they will be offered a baseline assessment and will be randomised only following completion of this.

#### Ethical approval

The study has been reviewed and given a favourable opinion by the London-Surrey Borders National Research Ethics Committee (REC Reference: 17/LO/04/45).

#### Intervention

The ACT/PE intervention will consist of ten groups, each running over ten consecutive weeks, followed by a booster session, 4 weeks later. Each group session will last for 2 h. The intervention will be delivered in addition to routine care (specialist care coordination, medication, practical and emotional support), and will be compared to routine care alone (TAU). After the 14-week assessment, individuals in the routine care condition will be offered the intervention.

The ACT/PE intervention will focus on psychoeducation approaches to understanding bipolar and mood disorders. Each session will focus on a different issue pertinent to bipolar disorder; including, mania, stress, sleep, depression, medication, communicating with friends and family, and relapse prevention. Each session will introduce ACT skills as a different way of responding to symptoms of bipolar disorder. The Balancing ACT groups are designed to be interactive with experiential exercises throughout and a commitment to work towards a valued goal, and the practice mindfulness exercises in between sessions.

We acknowledge that 14 weeks is a relatively short timeframe to assess change. For the pragmatic purposes of this study, because we are interested in the potential helpfulness of the intervention, rather than the longevity of the effects, we consider this time period appropriate. ACT has been shown to achieve change in brief interventions: previous studies from our research group [20] have shown significant change in 12 weeks during ACT interventions with clients with psychosis. This is longer than ACT interventions would normally be, but consistent with psychoeducation groups for people with bipolar disorder [6–8]. A future study can address maintenance of effects over time.

#### Therapists

We will train and supervise therapists within the service to deliver the intervention. Therapists will be trained to competence in delivery of the manualised intervention, will co-work with a member of the study team, and will be closely supervised by the trial coordinator.

### Measures

#### Outcome and timeline

Following informed consent from each participant, all outcomes will be assessed by a trained study researcher. Demographic/clinical characteristics (age, gender, ethnicity, diagnosis) will be assessed at baseline. Outcomes will be assessed at baseline (0 weeks), then at 10 weeks and 14 weeks post randomisation (Table 1).

**Table 1:** Balancing ACT: evaluating the effectiveness of psychoeducation and Acceptance and Commitment Therapy (ACT) groups for people with bipolar disorder: Study protocol for a pilot randomised controlled trial. List of measures

	Completed at:
Service user measures	
Brief Quality of Life in Bipolar Disorder (Brief QoL.BD: Michalak and Murray, 2009) [21]	1, 2, 3
Clinical Outcomes in Routine Evaluation Measure (CORE-10; Barkham et al., 2008) [25]	1, 2, 3
Valuing Questionnaire (VQ8; Smout et al., 2014) [27]	1, 2, 3
Acceptance and Action Questionnaire-II (AAQ-II; Bond et al., 2011) [28]	1, 2, 3
Southampton Mindfulness Questionnaire (SMQ; Chadwick et al., 2008) [29]	1, 2, 3
Bipolar Recovery Questionnaire (BRQ; Jones et al., 2013) [26]	1, 2, 3
Internal States Scale (ISS; Bauer, 1991) [24]	1, 2, 3
Researcher-rated measure	
Client Service Receipt Inventory (CSRI; Beecham and Knapp, 2001) [19] for the 12 months preceding and 3 months following baseline	1, 3
Service use (average/month) in preceding 12 months and following 3 months from baseline	
Sessional measures	
Brief Quality of Life in Bipolar Disorder (Brief QoL.BD: Michalak and Murray, 2009) [21]	Sessional

Key: Completed at: 1 = baseline (0 weeks), 2 = post therapy (10 weeks); 3 = post follow-up (14 weeks)

#### Trial and intervention feasibility parameters

The feasibility of the trial will be assessed using recruitment, randomisation and attrition rates. Intervention feasibility will be evaluated by treatment adherence and patient satisfaction.

#### Primary outcome

The proposed primary outcome will be psychological wellbeing assessed by the Brief Quality of Life in Bipolar Disorder (Brief QoL.BD) [21]. The Brief QoL.BD is well validated and routinely to assess quality of life in people with bipolar disorder [22, 23].

#### Secondary outcomes

The proposed secondary outcome of service use will be assessed from the medical records for the 12 months preceding entry to the study and the 3 months during the trial. We will calculate the number of admissions to hospital and contacts with the crisis/home treatment team as well as days under each of these services as an average per month. This is an indication of the severity of the presentation and the cost to the service of care.

#### Other clinical outcomes

Mood [24], distress recovery [25, 26] and psychological-change processes [27–29] will also be assessed at 0, 10 and 14 weeks. Mood will be assessed sessionally using an un-numbered Likert scale measuring 12 cm, rated from depression/down at one extreme, through normal at 6 cm to manic/high at the other extreme. We also aim to collect qualitative feedback from participants on their experiences of the Balancing ACT group and its impact on their recovery.

#### Service use and costs

We will approximate costs by counting average service use/month for the 12 months preceding randomisation [19], and the 3 months following randomisation. Service use variables will include number of psychiatric inpatient admissions, days as an inpatient, number of crisis team contacts, and days under the crisis team.

### Sample size

Resources allow us to recruit 36 participants to our study. Although this number is relatively small it will allow us to gain measures of trial feasibility (such as recruitment rates) and an estimation of the variance following dropout. Sample size estimates for effect size (ES) calculation may start from a total *N* of 24 [17]. However, ES calculated from small samples are sensitive to inflation [18] and we will take this inflation factor into account when conducting a power analysis for a larger trial.

### Randomisation

Randomisation will be carried out after consent to participate in the trial has been given and the baseline assessment has been carried out. Participants will be randomised through an independent web-based service provided by UKCRG registered King’s Clinical Trials Unit (Reg. No. 053). The randomisation procedure will employ methods to maintain pre-randomisation allocation concealment. We will stratify by site for logistical reasons, so that treatment cases are equitably allocated across sites.

### Blinding procedure

We will not be able to blind participants to treatment group. Similarly, the therapists cannot be blind to allocation as they will deliver the intervention. However, the research workers completing the outcome measurements will be blinded to treatment allocation. Should they be accidentally unblinded during the assessment, we will record which outcomes were completed blind and allocate a new assessor for any subsequent assessments. Post-randomisation assessors will work separately from the research and clinical teams to minimise the likelihood of unblinding. We will report any instances of unblinding in subsequent publications. The end of the trial will be defined as the last follow-up at 14 weeks. The trial statistician will also be blinded to treatment group for the analysis of clinical and functional outcomes.

### Data monitoring

Based on pre-pilot studies we do not anticipate risks to participant safety as a direct result of the study. We will not be conducting any interim data analysis and will not convene a separate Data Monitoring Committee. The trial may be prematurely discontinued by the sponsor, REC or chief investigator (with the agreement of the sponsor and the REC) on the basis of new safety information or for other reasons given by the sponsor or the REC. As the study is a small pilot, we will not convene a Trial Steering Committee. The study will be subject to the standard local and national governance frameworks of the sponsor, the Promoting Recovery Services and research coordination, the clinical research network and the REC.

### Data management

We will use paper assessment packs and enter data into electronic databases which include: (1) baseline demographics, (2) repeated clinical measures, (3) sessional therapy measures and (4) therapy delivery and adherence. Outcome assessments will be carried out by researchers who do not have access to therapy or feedback data. Data will be checked and cleaned against original paper copies and a final database returned to the statistician, who will combine with allocation data for analysis.

Patient data will be pseudonymised for the duration of the study and fully anonymised thereafter. Fully identifiable personal details will be kept on paper in a locked filing cabinet in a locked or occupied office; on secure NHS computers; and encrypted on password-protected computers. All trial data will be stored in line with the Data Protection Act [30].

The allocation database will be accessible only to the study randomiser (who will not conduct post-baseline assessments) and the trial statistician until the study is completed.

### Safety monitoring and adverse event reporting

We do not anticipate safety concerns arising as a direct result of the therapy, which is usually perceived as helpful by service users. However, we will monitor adverse events, defined as any deterioration in psychosocial functioning, irrespective of severity, carefully as one of our outcomes. All adverse events will be reported to the clinical team to ensure adequate care, and will be documented by the study team. Events considered by the participant, clinical service or research team to be unexpected and related to the study will be reviewed for seriousness. Serious adverse events that are related to the trial will be reported to the trial sponsor and the REC within 15 days.

### Statistical analysis

In accordance with the Consolidated Standards of Reporting Trials (CONSORT) principles, we will report all participant flow in the study. The aim of the analysis will be to describe the feasibility and acceptability of the trial ACT/PE intervention and estimate effect sizes for group differences in attitude, adherence, clinical and quality of life outcomes. Binary outcomes will be analysed using logistic regression, chi-square or Fisher’s exact test if expected values were small. Continuous outcome scores will be analysed using analysis of covariance (ANCOVA) models in which the mean group difference will be adjusted for baseline outcome scores. Clustering for patients within therapy groups will be accounted a random effect for cluster in the ANCOVA models. Effect sizes will be derived from standardised regression coefficients in which treatment group differences, adjusted for outcomes scores at baseline, will be divided by the standard deviation of the outcomes. Following Cohen’s guidelines, effect sizes will be treated as small (ES = 0.2), moderate (ES = 0.5) or large (ES = 0.8).

As a pilot study, the sample size may not give sufficient power to reject null hypotheses; however, we will derive inferential statistics on the intention-to-treat principle. As the sample size is small and regression assumptions may not be met and we will use robust methods, such as bootstrap resampling, to derive standard errors and bias-corrected accelerated confidence intervals. Missing data will be described per outcome and if possible mechanisms driving missingness accounted for in the statistical model under the assumption that data is missing at random and with sensitivity analyses. All statistical tests will be two-sided and confidence interval level set at 95%. STATA 14.1 (StataCorp, College Station, TX, USA) will be used to analyse the data.

## Discussion

**Fig. 2:**
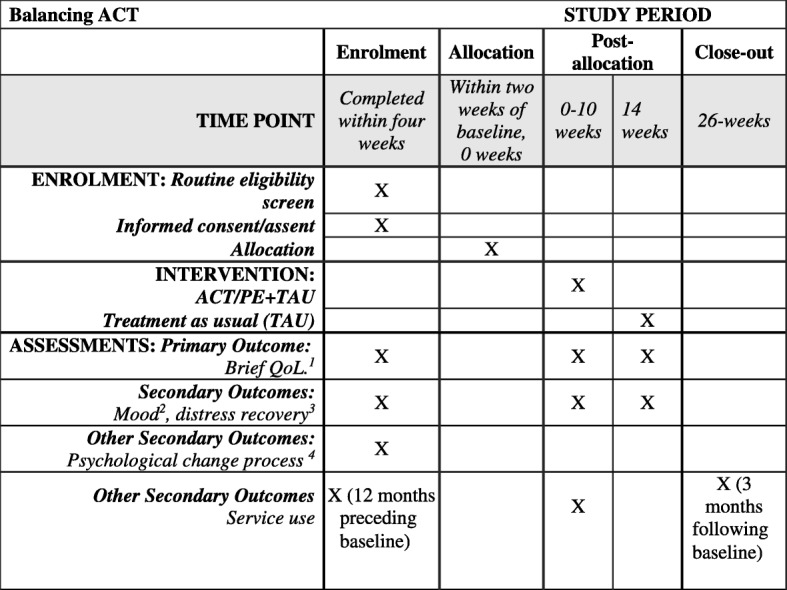
Balancing ACT: evaluating the effectiveness of psychoeducation and Acceptance and Commitment Therapy (ACT) groups for people with bipolar disorder: study protocol for a pilot randomised controlled trial. Schedule of enrolment, interventions and assessments

We have adhered to Standard Protocol Items: Recommendations for Interventional Trials (SPIRIT) [31, 32] guidance in devising and reporting our protocol (Fig. 2; SPIRIT Checklist with information sheets and consent forms for participants are included as an Additional file 1). The trial is funded until the end of July 2018. The study will be the first study of its kind examining ACT and psychoeducation groups for people with bipolar disorder and/or symptoms. Should the intervention prove feasible, acceptable and potentially helpful cost-effective addition to routine care, we will apply for funding for a larger-scale evaluation. If outcomes suggest that the intervention does not show promise in terms of potential clinical improvement, we will continue to refine the intervention, based on participant feedback, further consultation, and our process measures, before attempting a further pilot. Findings will be limited by the pilot nature of the study, including the small sample size, location in a single service and opportunistic recruitment strategy. The therapy will be compared to treatment as usual and not to an alternative psychological intervention, so any effects found may be non-specific. We are not assessing longevity of effects in this study: further research will be required to ascertain longer-term outcomes.

We plan to disseminate our findings via local academic and clinical networks, through conference presentation and publication. Authorship will be restricted to those making a substantial contribution to the specific publication.

### Status

Participants began to enter the trial in April 2017. The first participant was randomised on 10 May 2017. Recruitment will continue until end of October 2017. We anticipate that final primary outcome data will be collected by the end of February 2018.

## Additional file

Additional file 1: Standard Protocol Items: Recommendations for Interventional Trials (SPIRIT) 2013 Checklist: recommended items to address in a clinical trial protocol and related documents*. (DOC 121 kb)
